# Supplementary material for: Global hypo-methylation in a proportion of glioblastoma enriched for an astrocytic signature is associated with increased invasion and altered immune landscape
Source: eLife. 2022 Nov 22;11:e77335. doi: 10.7554/eLife.77335 (PMC9681209; doi:10.7554/eLife.77335)
Supplement: Figure 2—source data 1. [file elife-77335-fig2-data1.zip › Figure_2_source_data_1/Figure_2I_J/homerResults/motif7.similar.html]

motif7

## Information for motif7

G
T
A
C
G
A
C
T
C
A
T
G
A
C
T
G
A
C
T
G
C
T
A
G
A
T
C
G
G
A
T
C
G
T
A
C
T
A
G
C
A
T
G
C
C
A
G
T
A
C
T
G
A
C
G
T
A
T
C
G
A
T
G
C
G
C
A
T
A
T
C
G
C
G
A
T
A
T
C
G
G
A
C
T
A
C
T
G
A
T
G
C
A
T
G
C
A
T
G
C
G
C
A
T
A
T
C
G
G
A
C
T
A
C
T
G
C
G
T
A
A
T
G
C
A
G
T
C
G
A
T
C
G
A
T
C
G
A
T
C
A
T
G
C
G
C
A
T
A
T
G
C
G
A
C
T
A
G
C
T
G
A
T
C
A
G
T
C
G
T
A
C
A
C
G
T
C
A
T
G
  
Reverse Opposite:  

G
T
A
C
T
G
C
A
C
A
T
G
T
C
A
G
C
T
A
G
T
C
G
A
C
T
G
A
T
A
C
G
C
G
T
A
T
A
C
G
C
T
A
G
C
T
A
G
C
T
A
G
T
A
C
G
T
A
C
G
G
C
A
T
T
G
A
C
C
T
G
A
T
A
G
C
C
G
T
A
T
A
C
G
T
A
C
G
T
A
C
G
T
G
A
C
C
T
G
A
T
A
G
C
G
C
T
A
T
A
G
C
C
G
T
A
T
A
C
G
T
A
G
C
T
G
C
A
T
G
A
C
G
T
C
A
T
A
C
G
A
T
C
G
C
A
T
G
C
T
A
G
T
A
G
C
G
A
T
C
T
G
A
C
T
G
A
C
G
T
A
C
C
T
G
A
C
A
T
G
  

|  |  |
| --- | --- |
| p-value: | 1e-47 |
| log p-value: | -1.083e+02 |
| Information Content per bp: | 1.394 |
| Number of Target Sequences with motif | 51.0 |
| Percentage of Target Sequences with motif | 7.02% |
| Number of Background Sequences with motif | 2.0 |
| Percentage of Background Sequences with motif | 0.69% |
| Average Position of motif in Targets | 98.4 +/- 44.2bp |
| Average Position of motif in Background | 63.9 +/- 17.8bp |
| Strand Bias (log2 ratio + to - strand density) | 0.8 |
| Multiplicity (# of sites on avg that occur together) | 1.05 |
| Motif File: | file (matrix) reverse opposite |

### Similar de novo motifs found

|  |  |  |  |  |  |  |  |
| --- | --- | --- | --- | --- | --- | --- | --- |
| Rank | Match Score | Redundant Motif | P-value | log P-value | % of Targets | % of Background | Motif file |
| 1 | 0.622 | A C G T A T G C A G T C A G C T A G T C A T G C A C G T A C G T A G T C A T C G T A G C G A C T C A G T A C G T A T G C C G A T A T C G A G T C G A T C A G T C A G C T A G T C C G A T A C G T T A C G A C G T A G T C A G T C A G T C A G T C G A T C G A C T A G T C A G C T A G T C A G T C A G T C A G T C G A T C C A G T A T G C A G T C G T A C A T G C G T A C A G C T A T C G G A T C A T C G A C T G | 1e-34 | -79.870715 | 9.64% | 1.48% | motif file (matrix) |
